# Occupational Hearing Loss among Chinese Municipal Solid Waste Landfill Workers: A Cross-Sectional Study

**DOI:** 10.1371/journal.pone.0128719

**Published:** 2015-06-04

**Authors:** Yuewei Liu, Haijiao Wang, Shaofan Weng, Wenjin Su, Xin Wang, Yanfei Guo, Dan Yu, Lili Du, Ting Zhou, Weihong Chen, Tingming Shi

**Affiliations:** 1 Institute of Health Surveillance, Analysis and Protection, Hubei Center for Disease Control and Prevention, Wuhan, Hubei, 430079, China; 2 Key Laboratory of Environment and Health, Ministry of Education & Ministry of Environmental Protection, Wuhan, Hubei, 430030, China; 3 State Key Laboratory of Environmental Health (Incubating), Department of Occupational and Environmental Health, School of Public Health, Tongji Medical College, Huazhong University of Science and Technology, Wuhan, Hubei, 430030, China; Zhejiang University, CHINA

## Abstract

**Background:**

Occupational hearing loss is an increasingly prevalent occupational condition worldwide, and has been reported to occur in a wide range of workplaces; however, its prevalence among workers from municipal solid waste landfills (MSWLs) remains less clear. This study aimed to investigate the occupational hearing loss among Chinese MSWL workers.

**Methods:**

A cross-sectional study of 247 workers from 4 Chinese MSWLs was conducted. Noise and total volatile organic compounds (TVOCs) levels at worksites were determined. We conducted hearing examinations to determine hearing thresholds. A worker was identified as having hearing loss if the mean threshold at 2000, 3000 and 4000 Hz in either ear was equal to or greater than 25 dB. Prevalence of occupational hearing loss was then evaluated. Using unconditional Logistic regression models, we estimated the odds ratios (ORs) of MSWL work associated with hearing loss.

**Results:**

According to the job title for each worker, the study subjects were divided into 3 groups, including group 1 of 63 workers without MSWL occupational hazards exposure (control group), group 2 of 84 workers with a few or short-period MSWL occupational hazards exposure, and group 3 of 100 workers with continuous MSWL occupational hazards exposure. Both noise and TVOCs levels were significantly higher at worksites for group 3. Significantly poorer hearing thresholds at frequencies of 2000, 3000 and 4000 Hz were found in group 3, compared with that in group 1 and group 2. The overall prevalence rate of hearing loss was 23. 5%, with the highest in group 3 (36.0%). The OR of MSWL work associated with hearing loss was 3.39 (95% confidence interval [CI]: 1.28-8.96).

**Conclusion:**

The results of this study suggest significantly higher prevalence of hearing loss among MSWL workers. Further studies are needed to explore possible exposure-response relationship between MSWL occupational hazards exposure and hearing loss.

## Introduction

Occupational hearing loss is an increasingly prevalent occupational condition and occurs across many countries. It can be induced by several factors in workplace, such as noise, organic solvents, and other ototoxic substances. [1]. Noise is a common occupational hazard worldwide, and continues to be one of the largest causes of hearing loss [2,3]. In the United States, approximately 30 million people are occupationally exposed to hazardous noise every year. In 2009 alone, the US Bureau of Labor Statistics reported more than 21,000 hearing loss cases [4]. According to an analysis of adult hearing loss, China, Mongolia and South Korea have the highest proportion of sensorineural hearing loss attributable to occupational noise exposure all over the world [2].

Occupational hearing loss has been reported to occur in a wide range of workplaces, including printing, painting, manufacture of drug, foods and metal mining [5–8]. However, its prevalence among workers from municipal solid waste landfills (MSWLs) remains unclear. MSWL is a workplace to dispose of waste that cannot be economically reused, recycled or incinerated for energy recovery, and continues to be a necessary part of integrated solid waste management systems. In China, the total amount of municipal solid waste (MSW) collected and transported was 148 million tons in 2006, of which 91.4% was landfilled [9]. MSWL sites operate through the use of large machineries which dig the landfill, bring waste to the site, compact the waste and eventually begin the reclaiming process by covering the waste with clay and earth. Our previous study has demonstrated that there were several occupational hazards for workers in MSWLs, including noise, dust, toxic gases, heat, heavy metals, and total volatile organic compounds (TVOCs) [10].

As the main risk factor of hearing loss, noise in MSWLs is mainly generated by the large machineries and can be even over 90 dB that much higher than OSHA noise standard 1910.95 (85 dB) [10]. The risk of developing hearing loss is considered to be low at noise exposures below 85 dB (8-hour time-weighted average), but increases significantly when exposures rise above this level [11]. However, several studies have reported significantly increased risk of hearing loss even if the noise exposures were lower than 85 dB, especially when the noise exposures were combined with other occupational hazards that may also induce hearing loss [12–14]. In addition, certain chemicals, such as xylene and toluene that could exist in MSWLs have been reported to cause damage on the auditory system [15,16]. It is of importance to identify if the MSWL workers have more risk to develop occupational hearing loss.

Here, we carried out a cross-sectional study to conduct environmental monitoring at MSWL work sites, administrated audiometric testing and collected data through questionnaires of MSWL workers, aiming to investigate the occupational hazards, including noise and TVOCs, at MSWLs and evaluate the occupational hearing loss among MSWL workers.

## Methods

### Subjects and Data Collection

In this cross-sectional study, we investigated 4 Chinese MSWLs that were located in Shenzhen (Guangdong province), Shanghai, Harbin (Heilongjiang province) and Hangzhou (Zhejiang province) city, respectively. A total of 298 workers who had worked for at least 1 year were involved in our investigation. All these workers underwent and passed health examinations (including hearing examination) before their work at these landfills. We divided the subjects into 3 groups based on the specific work sites in the MSWLs where they mainly worked: group 1, general workers without occupational hazards (control group), including administration staff, engineering designers, guards, barbers, cooks, financial staff, cleaners, non-garbage truck drivers, etc.; group 2, workers with only a few or short-period occupational hazards associated with landfilling during their work shift, including environmental monitoring workers, laboratory assistants, road-menders, field directors, repairmen, technicians, safety managers, etc.; group 3, workers with continuous occupational hazards associated with landfilling, including bulldozer drivers, garbage truck drivers, excavating-machine operators, compacting machine operators, etc.

A specified questionnaire was implemented by trained investigators to collect data on demographic information (such as gender, year of birth, years of education), smoking status, drinking habit, etc. [10] Smoking status included nonsmoker and smokers that were defined as those who had smoked regularly for over 6 months. We used an additional questionnaire to collect each worker’s work history, including the current and all previous job titles with the corresponding start date and end date, work experience in other companies, as well as use of personal protective equipment, such as ear plugs, respirator, gloves, etc. All participants provided their written informed consent to participate in this study. This study and the consent procedure were approved by the Medical Review Ethics Committee of Hubei Center for Disease Control and Prevention.

### Noise Monitoring and Hearing Examination

Environmental noise levels of 4 MSWLs determined by work site environmental monitoring using portable sound level meter (AS-72, Danplex). The time-weighted noise levels for drivers or operators of large machineries were measured by attaching the Quest EDGE-4 Personal Noise Dosimeter during their work.

Before hearing examination, workers with local ear problems (such as ear injury, otitis media and tympanic membrane perforation) were excluded via local otoscopic examinations by a doctor. Workers eligible for hearing examination were asked for their work history, and if they had ever been diagnosed as hearing loss or deafness due to drugs, genetic disorders or any other risk factors. An audiometer (Itera, Madsen) with a middle ear analyzer (ZO 901, Madsen) was then used to measure the pure-tone air and bone conduction hearing thresholds at frequencies of 500, 1000, 2000, 3000, 4000 and 6000 Hz. The audiometric testing was conducted in a sound-isolated room and operated by a certificated occupational health doctor. All the candidate subjects were measured after a rest of at least 10 minutes in the morning prior to their work. The measured hearing thresholds were adjusted for gender and age based on the China National Standard “Diagnosis of occupational noise-induced deafness” (GBZ 49–2007). Under the current OSHA Standard (29 CFR 1904.10), a worker was defined as hearing loss if the audiogram identified a hearing threshold value of equal or greater than 25dB for the mean combined value of the 2000, 3000, and 4000 Hz in one or both ears [17,18].

### Statistical Analysis

Variables of age, years of education and duration of work were dichotomized based on their distributions in studied subjects. Continuous variables, such as noise level, for different groups of subjects with normal distribution and equal variance were described using means and standard deviations, and compared using Analysis of Variance (ANOVA) followed by the least significant difference (LSD) test for pair-wise comparisons. Continuous variables that did not met the conditions for ANOVA were described by median and interquartile range (IQR), and compared using Kruskal-Wallis test followed by Nemenyi test for pair-wise comparisons [19]. Categorical variables were compared using the Chi-square test or Fisher exact test.

Selected characteristics (including gender, age, years of education, smoking status, etc.), noise levels, result of audiometric testing and prevalence of hearing loss were compared among group 1, group 2 and group 3 of the study subjects. Odds ratios (OR) and the corresponding 95% confidence intervals (CIs) for different group as well as potential confounders associated with hearing loss were estimated using multivariate unconditional Logistic regressions. Because there was no female worker in group 3 of subjects, we also estimated ORs and 95%CIs by restricting the regression analysis in male workers only. All analyses were conducted with SAS version 9.1. All given *P* values are two-sided.

## Results

Of the 298 landfill waste landfill workers, all of them completed the questionnaire for demographic information; 2 (0.7%) of them did not finish the questionnaire for work history; 49 (16.4%) of them were not administrated audiometric testing; 2 (0.7%) of them suffered from significant hearing loss due to ototoxic drug use. No workers claimed any genetic risk for hearing loss. The 2 workers without work history did not finish audiometric testing neither; therefore, the 298 potential study subjects were reduced to 247 with a participant rate of 82.9%. Proportion of male workers for the 298 workers and the selected 247 workers were 81.0% and 80.6%, respectively; while the median age for the two populations were 39.0 and 38.0 years, respectively. Distributions and differences among subjects of selected characteristics were shown in Table 1. There were no female workers in group 3, and significantly more male workers in group 2 compared to group 1 (*P* < 0.05). Ratios of regularly smoking and drinking were significantly higher in group 3, compared to both group 1 and group 2 (*P* < 0.05). The education level of workers in group 3 was significantly lower than that in group 1 and group 2 (*P* < 0.05). These descriptive statistics and comparisons did not change materially for male workers only.

**Table 1:** Selected characteristics of the cross-sectional study of MSWL workers.

Characteristic	All workers				Male workers			
	n = 247	Group 1 (n = 63)	Group 2 (n = 84)	Group 3 (n = 100)	n = 199	Group 1 (n = 36)	Group 2 (n = 63)	Group 3 (n = 100)
Age, y, median (interquartile range)	38.0 (11.0)	41.0 (11.0)	37.0 (13.0)	38.0 (10.0)	38.0 (11.0)	39.0 (12.0)	36.0 (17.0)	38.0 (10.0)
Age > 38y, n (%)	122 (49.4)	35 (55.6)	39 (46.4)	48 (48.0)	94 (47.2)	20 (55.6)	26 (41.3)	48 (48.0)
Duration of education, y, median (interquartile range)	10.0 (3.0)	12.0 (6.0)	12.0 (5.0)	9.0 (2.0)^a^ ^,^ ^b^	9.0 (3.0)	11.0 (4.0)	12.0 (5.0)	9.0 (2.0)^a^ ^,^ ^b^
Duration of education > 10 y, n (%)	123 (49.8)	42 (66.7)	52 (61.9)	29 (29.0)^a^ ^,^ ^b^	93 (46.7)	23 (63.9)	41 (65.1)	29 (29.0)^a^ ^,^ ^b^
Regularly drinkers, n (%)	92 (37.3)	19 (30.2)	22 (26.2)	51 (51.0)^a^ ^,^ ^b^	92 (46.2)	19 (52.8)	22 (34.9)	51 (51.0)^b^
Smoker, n (%)	134 (54.3)	20 (31.8)	40 (47.6)	74 (74.0)^a^ ^,^ ^b^	134 (67.3)	20 (55.6)	40 (63.5)	74 (74.0)^a^
Duration of work, y, median (IQR)	10.0 (11.0)	8.5 (8.7)	8.2 (10.7)	11.0 (12.2)	9.4 (11.3)	8.2 (8.5)	6.9 (11.2)	11.0 (12.2)
Duration of work > 10 y, n (%)	116 (47.0)	27 (42.3)	36 (42.9)	53 (53.0)	92 (46.2)	14 (38.9)	25 (39.7)	53 (53.0)
Other landfill-related work (%)	17 (6.9)	7 (11.1)	2 (2.4)^a^	8 (8.0)	16 (8.0)	6 (16.7)	2 (3.2)	8 (8.0)

IQR, interquartile range.

^a^ *P* < 0.05 compared to group 1.

^b^ *P* < 0.05 compared to group 2.

Table 2 shows the noise levels by work sites of different groups of the workers. The mean noise level for 4 landfills was 66.1 dB with a range of 43.3 to 89.2 dB. The noise level at worksites of group 3 was significantly higher than that of group 1 and group 2 (*P* < 0.05), though there was no significant difference between group 1 and group 2. Of the 56 noise samples, 3 (5.4%) of them were over 85 dB, all of which were from group 3 (8.8%). The mean time-weighted noise exposure of bulldozer drivers and compacting machine operators were 95.1 dB and 91.1 dB, respectively. As shown in Table 3, we found significantly higher TVOCs level at worksites of group 3, compared to that of group 1 and group 2 (*P* < 0.05).

**Table 2:** Result of environmental noise monitoring by worksites of different groups of MSWL workers.

Work sites	Noise level (dB)						
	n	Mean^a^	SD	Median	Minimum	Maximum	> 85 dB (%)
Group 1	4	57.5	9.9	56.6	48.3	68.5	0.0
Group 2	18	60.7	10.0	61.3	47.3	81.3	0.0
Group 3	34	70.1^b^ ^,^ ^c^	12.6	71.9	43.3	89.2	8.8

^a^Compared by ANOVA test; *F* and *P* values are 4.99 and 0.010 respectively.

^b^ *P* < 0.05 compared to group 1.

^c^ *P* < 0.05 compared to group 2.

**Table 3:** Result of environmental TVOCs monitoring by worksites of different groups of MSWL workers.

Work sites	TVOCs level (mg/m^3^)					
	n	Mean	SD	Median^a^	Minimum	Maximum
Group 1	4	0.4	0.5	0.3	0	0.9
Group 2	8	0.7	0.5	0.6	0.01	1.7
Group 3	28	3.4	7.0	1.3^b^ ^,^ ^c^	0.22	36.8

^a^Compared by Kruskal-Wallis test.

^b^ *P* < 0.05 compared to group 1.

^c^ *P* < 0.05 compared to group 2.

Table 4 show the hearing thresholds of different groups of workers by frequencies of 2000, 3000 and 4000 Hz that were used in the evaluation of occupational hearing loss. For all frequencies, there was no significant difference for hearing thresholds between workers from group 1 and group 2 (*P* > 0.05); however, the hearing thresholds of group 3 was significantly higher than that of both group 1 and group 2 (*P* < 0.05). Similarly, the mean combined value of hearing thresholds at 2000, 3000 and 4000 Hz for workers from group 3 was significantly higher than that from group 1 and group 2 (*P* < 0.05).

**Table 4:** Result of audiometric testing of different groups of MSWL workers at frequencies of 2000, 3000 and 4000 Hz in either ear.

Frequency, Hz	Median (interquartile range), dB				
	All subjects (*n* = 247)	Group 1 (*n* = 63)	Group 2 (*n* = 84)	Group 3 (*n* = 100)	*P* value^a^
2000	14.0 (9.0)	13.0 (10.0)	14.0 (10.0)	15.0 (7.0)^b^ ^,^ ^c^	0.002
3000	13.0 (11.0)	11.0 (8.0)	12.0 (10.0)	15.0 (12.0)^b^ ^,^ ^c^	<0.001
4000	15.0 (16.0)	14.0 (12.0)	11.0 (15.0)	19.0 (21.5)^b^ ^,^ ^c^	<0.001
Average	14.0 (10.0)	12.3 (12.0)	12.5 (9.8)	16.7 (12.0)^b^ ^,^ ^c^	<0.001

^a^Compared by Kruskal-Wallis test among group 1, group 2 and group 3.

^b^ *P* < 0.05 compared to group 1.

^c^ *P* < 0.05 compared to group 2.

Based on the OSHA Standard, there were 58 hearing loss cases in all investigated workers, resulting in a prevalence rate of 23.5%. All these hearing loss cases were sensorineural hearing loss cases. The number of hearing loss cases in group 1, group 2 and group 3 were 10 (prevalence rate: 15.9%), 12 (14.3%), and 36 (36.0%) respectively. The number and prevalence of hearing loss by selected characteristics in different groups of the subjects was showed in Table 5. Significant trends of increased prevalence of hearing loss from group 1 to group 3 were observed by all characteristics (*P* < 0.05) except for worker with experience of smoking and other landfill-related work.

**Table 5:** Hearing loss based on OSHA standard by selected characteristics in different groups of MSWL workers.

Characteristic	Hearing loss, n (%)			
	Group 1 (n = 63)	Group 2 (n = 84)	Group 3 (n = 100)	*P* trend^a^
Overall	10 (15.9)	12 (14.3)	36 (36.0)^b^ ^,^ ^c^	
Gender				
Male	8 (22.2)	9 (14.3)	36 (36.0)^c^	0.02
Female	2 (7.4)	3 (14.3)	N.A.	N.A.
Age, y				
≤ 38	4 (14.3)	6 (13.3)	18 (34.6)^b^ ^,^ ^c^	0.02
> 38	6 (17.1)	6 (15.4)	18 (37.5)^b^	0.02
Duration of education, y				
< 10	2 (9.5)	5 (15.6)	24 (33.8)^b^	0.01
≥ 10	8 (19.1)	7 (13.5)	12 (41.4)^b^ ^,^ ^c^	0.05
Drinking				
No	7 (15.9)	10 (16.1)	18 (36.7)^b^ ^,^ ^c^	0.01
Yes	3 (15.8)	2 (9.1)	18 (35.3) ^c^	0.04
Smoking status				
Nonsmoker	6 (14.0)	7 (15.9)	14 (53.9)^b^ ^,^ ^c^	0.005
Smoker	4 (20.0)	5 (12.5)	22 (29.7)^c^	0.12
Duration of work, y				
≤ 10	6 (16.7)	7 (14.6)	14 (34.0)^c^	0.04
> 10	4 (14.8)	5 (13.9)	20 (37.7)^b^ ^,^ ^c^	0.01
Other landfill-related work				
No	8 (14.3)	12 (14.6)	32 (34.8)^b^ ^,^ ^c^	0.002
Yes	2 (28.6)	0 (0.0)	4 (50.0)	0.37

N.A., not applicable.

^a^Cochran-Armitage trend test.

^b^ *P* < 0.05 compared to group 1.

^c^ *P* < 0.05 compared to group 2.

The multivariate unconditional Logistic regressions gave estimated odds ratios and the corresponding 95% CIs for groups and potential confounders, including different groups, gender, age, years of education, drinking, smoking status, duration of work and other landfill-related work (Table 6). Hearing loss was found to be significantly higher in group 3 in the models including either all subjects (OR: 3.39; 95% CI: 1.28–8.96) or male workers only (2.97; 1.09–8.10).

**Table 6:** Prevalence and odds ratios of hearing loss by selected characteristics in MSWL workers.

Characteristic	All workers		Male workers only	
	Prevalence of hearing loss	Odds Ratio	Prevalence of hearing loss	Odds Ratio
Groups				
Group 1	10 (15.9)	1.00	8 (22.2)	1.00
Group 2	12 (14.3)	0.90 (0.34–2.35)	9 (14.3)	0.66 (0.22–2.00)
Group 3	36 (36.0)	**3.39 (1.28–8.96)**	36 (36.0)	**2.97 (1.09–8.10)**
Gender				
Female	5 (10.4)	1.00	N.A.	N.A.
Male	53 (26.6)	2.98 (0.93–9.57)	53 (26.6)	N.A.
Age, y				
≤ 38	28 (22.4)	1.00	26 (24.8)	1.00
> 38	30 (24.6)	1.40 (0.71–2.76)	27 (28.7)	1.44 (0.70–2.98)
Duration of education, y				
≤ 10	31 (25.0)	1.00	29 (27.4)	1.00
> 10	27 (22.0)	1. 38 (0.69–2.79)	24 (25.8)	1.49 (0.70–3.15)
Drinking				
No	35 (22.6)	1.00	30 (28.0)	1.00
Yes	23 (25.0)	0.79 (0.40–1.58)	23 (25.0)	0.77 (0.39–1.54)
Smoking status				
Nonsmoker	27 (23.9)	1.00	22 (33.9)	1.00
Smoker	31 (23.1)	0.47 (0.23–1.00)	31 (23.1)	0.48 (0.23–1.00)
Duration of work, y				
≤ 10	29 (22.1)	1.00	27 (25.2)	1.00
> 10	29 (25.0)	1.00 (0.52–1.94)	26 (28.3)	0.94 (0.46–1.91)
Other landfill-related work				
No	52 (22.6)	1.00	47 (25.7)	1.00
Yes	6 (35.3)	1.75 (0.58–5.33)	6 (37.5)	1.81 (0.58–5.61)

N.A., not applicable.

## Discussion

The results of our study suggested significantly higher prevalence of hearing loss among MSWL workers. Noise and TVOCs might be potential risk factors for MSWL workers to develop hearing impairment, although their causal relationship needs to be further investigated by conducting quantitative exposure assessment and exposure-response analyses. Nonetheless, our data provided a primary result regarding the prevalence of hearing loss among MSWL workers.

The hearing loss among MSWL workers may be mostly attributable to the high noise exposure. There are several possible explanations to address this. One is the higher noise level for MSWL workers. The large machineries that were used to dig, transport and compact landfills can generate noise level higher than 85 dB (group 3). The result of environmental noise monitoring showed that the mean noise level was 70.1 dB which was significantly much higher than that in work sites of group 1 (57.5 dB) and group 2 (60.7 dB). The other reason is the continuity of noise exposure (group 3). The exposure pattern of group 3 was continuously exposed to a relatively constant level of noise during work time. Continuous noise exposure throughout the workday and over years can be more damaging than interrupted exposure to noise that permits the ear to have a rest period (group 2) [11]. The last potential explanation is the poor use of personal hearing protection. Among the studied subjects, only 3 (1.2%, in group 2) workers wore ear plugs during their work, and none of the subjects in group 3 wore any ear protection equipment despite frequent exposure to noise levels over 85 dB. Based on the China National Standard “Hygienic standards for the design of industrial enterprises” (GBZ 1–2010), industrial enterprises should try to reduce occupational noise to lower than 85 dB at workplace, or workers should use personal protective equipment, such as ear plugs. It is important for the studied landfills to improve waste processing to lower the worksite noise, provide workers with personal protective equipment and train them how to correctly use it. Besides, according to the China National Standard “Technical specifications for occupational health surveillance” (GBZ 188–2014), workers with exposure to occupational noise should undergo health examinations before and after the work, and undergo health examinations every year for noise level over 85 dB. The studied landfills need to follow this standard to conduct regular health surveillance to protect their workers from developing occupational hearing loss.

Meanwhile, other occupational hazards might have contributed to the higher prevalence of hearing loss among MSWL workers. Besides noise, our previous study showed that the main occupational hazards also included respirable dust, heat, ozone, ammonia, methane, hydrogen sulfide and TVOCs [10]. A study investigating the characteristics of VOCs emitted by open landfills receiving municipal solid waste reported high concentrations of toluene, xylene and trichloroethylene [20]. These chemicals have been reported to have neurotoxic, ototoxic effects [15]. Mohammadi *et al*. found that combined exposure to mixed organic solvents (including benzene, toluene, xylene, etc.) and noise could exacerbate hearing loss, especially high-frequency (average hearing threshold > 25 dB at 3, 4, 6, and 8 kHz) hearing loss, in workers [21]. By evaluating the hearing of painting workers with exposed to noise a mixture of organic solvents (including toluene vapor, xylene vapor, etc.) at concentrations anticipated as safe, Metwally *et al*. found elevated hearing impairment compared to workers exposed to noise only, suggesting that the solvent exposure contributed process of hearing loss [12]. In this study, our results showed significantly higher TVOCs at MSWL worksite, and some of them were much higher than that of the OSHA standard; therefore, hearing impairment by exposure to TVOCs should not be neglected when considering the cause of hearing loss among MSWL workers.

Various studies have shown that age, smoking and some other factors are associated with higher prevalence of hearing loss [12,22,23]. In the present study, we used stratified analyses by these confounders to learn the prevalence of hearing loss, and obtained similar results. The prevalence of hearing loss in MSWL workers (group 3) was significantly higher than that in group 1 and group 2, although a few of them did not reach statistically significance, partly because of their small sample sizes. From multivariate Logistic regressions, our study did not suggest any association between hearing loss among MSWL workers and selected characteristics, including gender, age, years of education, smoking and drinking status, duration of work and other landfill-related work. Although the odds ratio of age and duration of work were much larger than 1, they did not show statistical significance. Because there were no female workers in group 3 of the subjects, we also conducted multivariate Logistic regression among male workers only. The result showed similar trends, with slightly elevated odds ratio for group 3 compared with the odds ratio estimated by all the subjects.

Several limitations should be addressed for our study. One is that the MSWLs in our study did not have sufficient data on previous occupational noise exposure. In the analysis, we used our monitoring data to evaluate noise level, and assumed that the noise level had not changed overtime. This might have led to inaccuracy as regards to the noise exposure; however, the noise levels for MSWL workers, especially drivers of large machineries, were not likely to change dynamically. Second, the sample size is relatively small. Unlike other studies [23], odds ratios of age in relation to hearing loss was much large than 1, but did not reach statistically significance. Nevertheless, significantly elevated odds ratio for group 3 was observed, despite of a wide confidence interval. Finally, though we tried our best to exclude hearing loss cases caused by non-occupational risk factors, we did not collect sufficient information on previous history of diabetes and high blood pressure and take them into consideration.

## Conclusion

The present study demonstrated increased prevalence occupational hearing loss among MSWL workers. Possible reasons may include exposure to noise and TVOCs. Further studies needs to be conducted to better understand associations between occupational risk factors and hearing loss. Hearing conservation program should be considered to implement in workplaces of MSWLs. Health promotion should also be conducted to help MSWL workers protect themselves from excess noise exposure, such as regular health examinations and personal protective equipment use.
